# Molecular Mechanisms of Synaptotoxicity and Neuroinflammation in Alzheimer’s Disease

**DOI:** 10.3389/fnins.2018.00963

**Published:** 2018-12-14

**Authors:** Mikael Marttinen, Mari Takalo, Teemu Natunen, Rebekka Wittrahm, Sami Gabbouj, Susanna Kemppainen, Ville Leinonen, Heikki Tanila, Annakaisa Haapasalo, Mikko Hiltunen

**Affiliations:** ^1^Institute of Biomedicine, University of Eastern Finland, Kuopio, Finland; ^2^Institute of Clinical Medicine – Neurosurgery, University of Eastern Finland, Kuopio, Finland; ^3^Department of Neurosurgery, Kuopio University Hospital, Kuopio, Finland; ^4^Unit of Clinical Neuroscience, Neurosurgery, University of Oulu, Oulu, Finland; ^5^Medical Research Center, Oulu University Hospital, Oulu, Finland; ^6^A.I. Virtanen Institute for Molecular Sciences, University of Eastern Finland, Kuopio, Finland

**Keywords:** Alzheimer’s disease, microglia, neuroinflammation, synaptotoxicity, triggering receptor expressed on myeloid cells 2

## Abstract

Alzheimer’s disease (AD) is the most common neurodegenerative disorder, which is clinically associated with a global cognitive decline and progressive loss of memory and reasoning. According to the prevailing amyloid cascade hypothesis of AD, increased soluble amyloid-β (Aβ) oligomer levels impair the synaptic functions and augment calcium dyshomeostasis, neuroinflammation, oxidative stress as well as the formation of neurofibrillary tangles at specific brain regions. Emerging new findings related to synaptic dysfunction and initial steps of neuroinflammation in AD have been able to delineate the underlying molecular mechanisms, thus reinforcing the development of new treatment strategies and biomarkers for AD beyond the conventional Aβ- and tau-targeted approaches. Particularly, the identification and further characterization of disease-associated microglia and their RNA signatures, AD-associated novel risk genes, neurotoxic astrocytes, and in the involvement of complement-dependent pathway in synaptic pruning and loss in AD have set the outstanding basis for further preclinical and clinical studies. Here, we discuss the recent development and the key findings related to the novel molecular mechanisms and targets underlying the synaptotoxicity and neuroinflammation in AD.

## Introduction

Alzheimer’s disease (AD) is the main cause of dementia accounting for over 70% of dementia cases world-wide (Prince et al., 2016). AD is clinically manifests as memory impairment and executive dysfunction interfering with daily activities, rendering the affected incapable of independent living (Scheltens et al., 2016). The defining neuropathological features of AD consist of accumulation extracellular amyloid plaques and intraneuronal neurofibrillary tangles (NTFs), comprised of densely packed amyloid-β (Aβ) peptides and phosphorylated tau, respectively (Scheltens et al., 2016). According to prevailing amyloid cascade hypothesis, accumulation of these proteins are considered to follow a temporal sequence, with Aβ accumulation triggering a cascade of events comprising NFT formation, synaptotoxicity, mitochondrial dysfunction, and neuroinflammation due to aberrant activation of microglia and astrocytes (De Strooper and Karran, 2016). Aβ is derived from the amyloid precursor protein (APP) via sequential proteolytic cleavage by β- and γ-secretases (Zhang et al., 2011). Despite the accumulation of Aβ being a hallmark of AD, the physiological functions of APP remain largely undetermined with previous research highlighting potential roles for APP in processes, such as neurite outgrowth, axonal protein trafficking, transmembrane signal transduction, and calcium metabolism (Van Der Kant and Goldstein, 2015). Therefore, it is expected that lowering the Aβ levels is sufficient to slow down the disease process in AD, if started early enough. This concept is supported by the identification of a protective variant in *APP* gene (APP A673T), which reduces significantly the risk of AD and associates with decreased plasma levels of Aβ in individuals carrying protective *APP* variant (Jonsson et al., 2012; Martiskainen et al., 2017). However, the reported Aβ-targeted trials in AD patients to date have not been successful, addressing the need to test alternative therapeutic approaches beyond Aβ focusing on other key early events, including synaptic dysfunction, hyperphosphorylation and aggregation of tau or the initial steps of neuroinflammation. In this review, we summarize and discuss the recent development and the key findings related to the novel molecular mechanisms and targets underlying synaptotoxicity and neuroinflammation in AD.

## Aβ-Induced Synaptic Dysfunction and Synaptotoxicity

**FIGURE 1:**
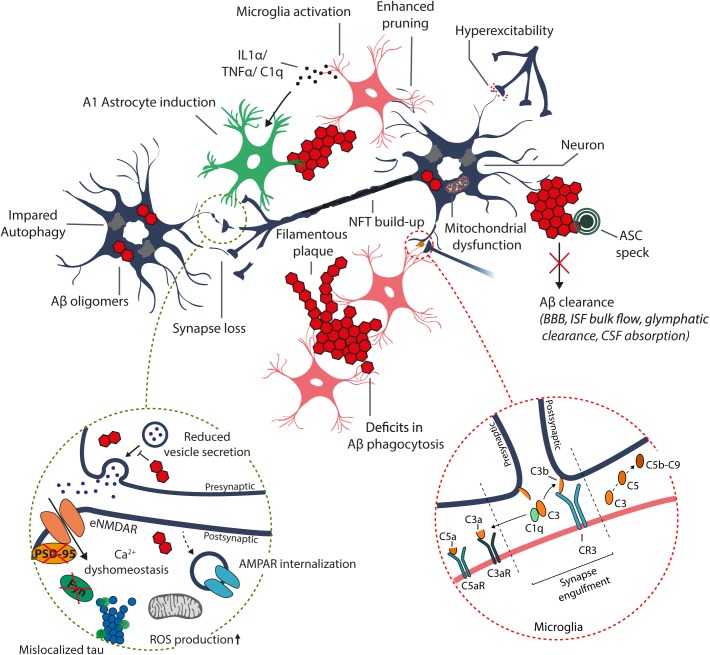
Schematic representation of the suggested alterations induced by excess accumulation of Aβ in the brain. Aβ oligomers and plaque-like structures build up due to increased amyloidogenic processing of the APP and/or disrupted Aβ clearance systems, including enzymatic degradation (autophagy-lysosome degradation), transport across the blood–brain barrier (BBB), bulk flow of interstitial fluid (ISF) and glymphatic clearance, and absorption to the cerebrospinal fluid (CSF) and further into the circulatory and lymphatic systems. The increase in intracellular Aβ oligomer species may disrupt synaptic transmission and induce postsynaptic hyperexcitability, resulting in Ca2+ dyshomeostasis, increased mitochondrial reactive oxygen species (ROS) production, and internalization of α-amino-3-hydroxy-5-methyl-4-isoxazolepropionic acid receptors (AMPARs), leading to synaptic depression (dashed circle). Furthermore, accumulation of intracellular Aβ results in hyperphosphorylation and mislocalization of tau to postsynaptic sites, which disrupts tau functionality and alters recruitment of essential proteins required for synaptic potentiation, such as FYN and post-synaptic density protein 95 (PSD-95). Moreover, the increased Aβ burden results in aberrant activation and dysfunction of immune cells (astrocytes and microglia), leading to excess production of various inflammatory cytokines and chemokines, and impairment in functions, including Aβ phagocytosis.

In AD, synapses are considered the earliest site of pathology, and reduced synaptic activity is found to be the best pathological correlate of cognitive impairment in AD (Coleman and Yao, 2003). Furthermore, several studies have demonstrated that pathological elevation of Aβ levels reduces glutamatergic synaptic transmission, resulting in synapse loss (Snyder et al., 2005; Tackenberg et al., 2013; Tu et al., 2014). Despite the pathological functions of Aβ, intermediate Aβ levels are considered necessary in presynaptic regulation (Figure 1; Abramov et al., 2009). Also, synaptic activity modulates Aβ production (Parihar and Brewer, 2010). Increased synaptic activity is thought to enhance endocytosis of APP, enabling increased Aβ production and potentiating Aβ secretion (Cirrito et al., 2008). Consequently, these relatively small increases in Aβ abundance have been shown to potentiate synaptic vesicle recycling, potentially through activation of α7-nicotinic acetylcholine receptor (Lazarevic et al., 2017). However, excess accumulation of Aβ has been shown to promote excitotoxicity, triggering long-term depression (LTD) in synapses, potentially as a result of postsynaptic N-methyl-D-aspartate receptor (NMDAR) desensitization, NMDAR and α-amino-3-hydroxy-5-methyl-4-isoxazolepropionic acid receptor (AMPAR) internalization, and overstimulation of extrasynaptic NMDARs (eNMDARs; Coleman and Yao, 2003). Postsynaptic activation is tightly regulated by the number of active plasma membrane-localized NMDARs and AMPARs. NMDAR activation has a pivotal role in synaptic transmission, as it is capable of inducing either LTP or LTD, by regulating the extent of intracellular calcium (Ca^2+^) levels and thus activation of downstream pathways related to AMPAR trafficking (Lüscher and Malenka, 2012). Induction of LTP requires activation of NMDARs, leading to a large increase in postsynaptic Ca^2+^ levels and activation of downstream events, including re-localization of AMPARs to the plasma membrane. In contrast, LTD induction requires NMDAR internalization, activation of extrasynaptic NMDARs and modest increases in Ca^2+^ levels (Lüscher and Malenka, 2012).

Emerging evidence suggests that Aβ-induced excitotoxicity results from aberrant stimulation of NMDARs, potentially due to the impairment of the regulation of glutamate levels or plasma membrane NMDAR composition (Figure 1; Falcon and Gentleman, 2007; Matos et al., 2008; Li et al., 2011; Ferreira et al., 2012). Excess glutamate in the synaptic cleft can potentially desensitize NMDARs, not allowing sufficient Ca^2+^ influx and LTP induction (Shankar et al., 2007). Additionally, increased glutamate levels may result in overstimulation of eNMDARs, triggering aberrant activation of redox-mediated events as well as Ca^2+^ dyshomeostasis, among other downstream events (Hardingham and Bading, 2010). Consequently, increased Aβ levels have been associated with increased AMPAR internalization and thus enhanced LTD (Figure 1; Guntupalli et al., 2016). The relevance of NMDAR overstimulation in AD is supported by studies reporting that blocking of NMDA overstimulation with partial NMDAR antagonists alleviates neurodegeneration in various animal models (Decker et al., 2010; Rönicke et al., 2011). This partial NMDAR antagonism is suggested to account for the beneficial clinical effect of memantine in AD (Danysz et al., 2000). As mentioned previously, increased Aβ levels potentially initiate a pathway leading to tau-mediated synaptic dysfunction. Under normal conditions, tau is translocated from the dendritic shaft to postsynaptic compartments under synaptic activation. Concomitant to postsynaptic translocation of tau, Fyn kinase is recruited to the postsynaptic compartment, which phosphorylates NMDAR, facilitating the interaction between PSD-95 and NMDAR and NMDAR stabilization (Ittner et al., 2010; Frandemiche et al., 2014). This stabilization of NMDARs, are potentially necessary for LTP-associated synaptic potentiation (Frandemiche et al., 2014). However, dissociation of the Tau/Fyn/PSD-95/NMDAR complex seems to be equally crucial as this complex is suggested to mediate Aβ-related excitotoxicity (Ittner et al., 2010). Interestingly, increase in Aβ levels potentiates the localization of tau, Fyn, and PSD-95 to postsynaptic sites independent of synaptic activation. In turn, translocation of Tau, Fyn, and PSD-95 is impaired by increased Aβ levels during synaptic activation, potentially disrupting LTP-required synaptic potentiation (Frandemiche et al., 2014). An increase in oligomeric Aβ has also been shown to reduce tau threonine 205 (T205) phosphorylation, which is a key tau phosphorylation site regulating the stability of the Tau/Fyn/PSD-95/NMDAR complex (Ittner et al., 2016). Under normal synaptic activation, post-synaptically localized tau is phosphorylated on threonine 205 by p38γ, dissociating the Tau/Fyn/PSD-95/NMDAR complex. In turn, p38γ-mediated tau phosphorylation is largely decreased upon excess Aβ exposure (Ittner et al., 2016). Collectively, Aβ is essential for synaptic transmission, but its accumulation leads to impairment in regulation of LTP/LTD induction, potentially through mechanisms related to NMDAR activation, NMDAR and AMPAR trafficking, and tau translocation and phosphorylation in dendrites.

## Microglia in the AD-Associated Neuroinflammation and Synaptic Elimination

Neuroinflammation in AD is thought to be primarily driven by microglial cells. Aβ oligomers and fibrils are capable of priming microglial cells through interactions with various receptors, which enhance the production of inflammatory cytokines and chemokines [interleukin-1, interleukin-6, tumor necrosis factor α (TNF-α), C1q etc.] and make microglia more susceptible to secondary stimuli, promoting microglial activation (Heppner et al., 2015). In AD, aggregation of Aβ results in chronic activation of primed microglia, enhancing production of inflammatory cytokines and chemokines, which in turn further aggravate primed microglia (El Khoury et al., 2003; Stewart et al., 2010). More specifically, recent findings highlight a critical role for the activation of the NACHT-, Leucine rich repeat- and pyrin-domain-containing protein 3 (NLRP3) inflammasome, which in addition to promoting pro-inflammatory cytokine release also induces the assembly of adaptor protein apoptosis-associated speck-like protein containing a CARD (ASC) to helical fibrillar structures (Venegas et al., 2017). These structures, better known as ASC specks, accelerate Aβ aggregation and spreading, and potentially sustain the ongoing immune response via activation of surrounding immune cells (Figure 1). This vicious cycle impairs microglial functions, affecting astrocyte, oligodendrocyte and neuron functions, in addition to potentially promoting tau pathology and ultimately neurodegeneration (Heppner et al., 2015). Consequently, prolonged activation of microglia can result in microglial cells acquiring a detrimental phenotype, which is thought to be irreversible (Miller and Streit, 2007). Additionally, a role for microglia in postnatal synaptic pruning has been demonstrated, with complement proteins potentially playing a pivotal role in regulating the extent of pruning (Paolicelli et al., 2011; Stephan et al., 2012). Complement protein C1q has been shown to be upregulated up to 80-fold in human AD brains, and to activate the complement cascade upon detection of Aβ oligomers (Kolev et al., 2009; Figure 1). In AD mouse models, complement system is initiated via the classical, lectin or alternative pathways, leading to cleavage of C3 with subsequent downstream effects on microglia-specific synapse engulfment, activation of microglial inflammatory signaling and lysis of synapses via formation of C5b-C9 membrane attack complex (Hong et al., 2016). This results in enhanced microglia-mediated synapse loss, further driving neurodegeneration. Importantly, knockout of C3 protects against neurodegeneration in aged plaque-rich transgenic mice (Shi et al., 2017). As a further support for the involvement of complement cascade in the pathological processes relevant for AD, knock-out of CD59 protein, which inhibits the C5b-C9 membrane attack complex, aggravated the tau pathology in aged P301L mutant tau transgenic mice (Britschgi et al., 2012). Taken together, microglial cells play a major role in different facets of AD-associated neuroinflammation and synaptic elimination processes. Further, specific molecular determinants in microglia, such as TREM2, are central for the function and activity of microglia in these processes.

## Microglial Activation Stages are Closely Linked to TREM2

**FIGURE 2:**
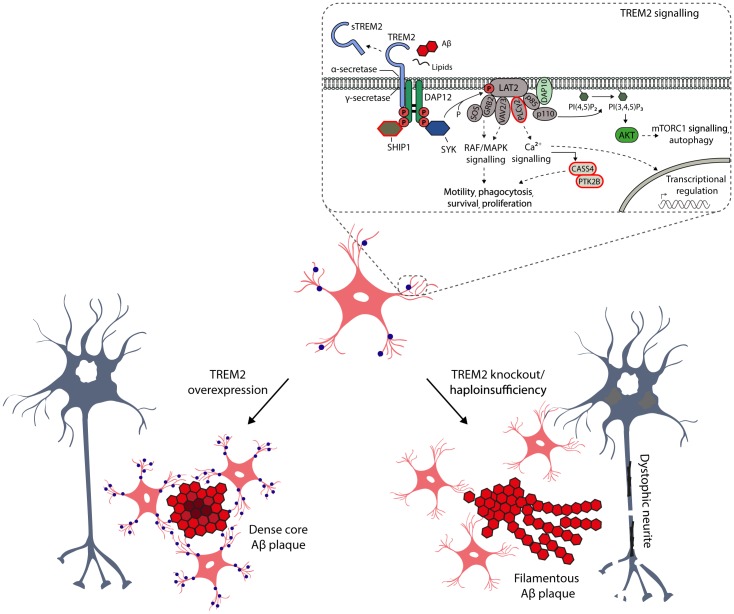
Schematic representation of the key role of TREM2 in the microglia functions and activation. TREM2 is a cell surface receptor in microglia, which by interacting with activating adaptor protein DAP12 initiates a signaling cascade leading to enhancement of pathways regulating phagocytosis, motility, survival and proliferation of microglia. TREM2 binds several ligands, such as phospholipids, lipoproteins, heparin sulfate proteoglycans, as well as apoptotic neurons and oligomeric Aβ. Importantly, TREM2 is also a target for α- (ADAM10) and γ-secretase-mediated cleavage, resulting in the release of soluble TREM2 (sTREM2) from the ectodomain into the interstitial and further to cerebrospinal fluid. Therefore, sTREM2 is actively been evaluated in CSF as a potential early biomarker for neurodegenerative diseases (Suarez-Calvet et al., 2016). The CSF levels of sTREM2 are increased in the asymptomatic individuals carrying the causative mutations for AD, suggesting that the activation of microglia takes place several years before onset of the disease, but importantly after the amyloidosis and neuronal injury have already emerged. There are several AD-associated non-synonymous variants in *TREM2*, which are located at the ectodomain of the receptor. Apart from these variants, recent GWAS studies have identified novel AD-associated risk genes, which are expressed selectively or preferentially in microglia (circled in red) and which are at least partially linked to TREM2 signaling pathways. Studies conducted in genetically manipulated TREM2 mice have provided compelling evidence that the TREM2 is the key activator of microglia upon Aβ-induced neuroinflammation, affecting the microglial barrier, metabolic fitness and autophagosomal activity of microglia as well as compaction of Aβ plaques and formation of dystrophic neurites.

It has become evident that specific microglial checkpoint mechanisms, such as seclusion from circulation, soluble restrain factors, cell–cell interactions and transcriptional regulators restrain the microglial immune activation throughout life, thus promoting the homeostatic functions of microglia in central nervous system (CNS; Deczkowska et al., 2018). Consequently, these checkpoint mechanisms may limit the capability of microglia to protect CNS when vigorous immune response is needed. Interestingly, a study employing transcriptional single-cell sorting in a mouse model of AD revealed a novel microglia type associated with neurodegenerative diseases (DAM; Keren-shaul et al., 2017). DAM was shown to be regulated through a two-step activation mechanism. In the first step, which was TREM2 independent, increased expression of specific set of genes, such as TREM2-signaling adaptor protein (*DAP12/TYROBP*) and *APOE* was detected, while the homeostatic microglial markers, such as *CX3CR1* and *P2RY12*, showed decreased expression. The second step involved a switch toward increased expression of specific genes, such as *TREM2*, *LPL*, *CST7*, and *CLEC7A*, which was dependent on the TREM2 status (Keren-shaul et al., 2017). Importantly, the identified DAM genes are closely linked to phagocytic and lipid metabolism pathways, and DAMs are localized in the vicinity of Aβ plaques in plaque bearing transgenic mice and post-mortem AD samples. Apart from AD, DAMs function as phagocytic cells in other neurodegenerative diseases, such as amyotrophic lateral sclerosis (ALS), but also in the aging (Keren-shaul et al., 2017). At the same time, it was reported that TREM2-APOE pathway drives transcriptional phenotype of dysfunctional microglia in neurodegenerative diseases (Krasemann et al., 2017). More specifically, expressional induction of APOE upon aging and disease progression in mouse models of ALS, multiple sclerosis (MS) and AD was instrumental for the development of the specific molecular signature of common disease-related microglia alongside with the suppression of tolerogenic TGFβ signaling (Krasemann et al., 2017). Both APOE-induced and -repressed gene sets, including some of the key microglia-specific transcription factors, were identified upon injection of apoptotic neurons to the cortex and hippocampus of wild-type and APOE knockout mice (Krasemann et al., 2017). Again, the APOE-mediated regulation of microglia was found to be TREM2-dependent as the genetic targeting of TREM2 (TREM2 knockout) supressed the APOE pathway and restored the homeostatic microglia in mouse models of AD and ALS (Krasemann et al., 2017). Further support for the seminal role of TREM2 in microglia was obtained when human TREM2 gene dosage was increased in the 5xFAD mouse model of AD (Lee et al., 2018). Elevated levels of TREM2 reduced the expression of DAM-specific genes alongside with the mitigation of the AD-related pathology in the brain of 5xFAD mice. Importantly, increased levels of TREM2 rescued the down-regulation of neuronal genes and reduced phagocytic activity of microglia observed in 5xFAD mice, as well as reduced neuritic dystrophy and improved memory performance. In line with these findings, the activation or up-regulation of TREM2 enhanced the phenotypic conversion of microglia and decreased the number of apoptotic neurons in mice with acute brain ischemia (Zhai et al., 2017). Collectively, these data indicate that microglial activation stages and the RNA signature of specific DAM genes are closely linked to TREM2 in AD and in other neurodegenerative diseases (Figure 2).

## TREM2 as a Master Regulator of Microglia Functions Upon AD-Associated Neuroinflammation

Given the seminal role of TREM2 in the DAM, it is crucial to distinguish the underlying molecular mechanisms of this myeloid cell-specific membrane receptor in the physiological as well as AD-associated pathological conditions. It is well-established that TREM2 binds different extracellular ligands, such as phospholipids, lipoproteins, heparin sulfate proteoglycans, and apoptotic neurons, which activates intracellular signaling pathways affecting the phagocytotic activity, migration, survival and proliferation of microglia (Figure 2; Yeh et al., 2016; Park et al., 2018). Moreover, microglia have been suggested to take part in extracellular Aβ phagocytosis, and mutations in *TREM2* gene affecting TREM2 shedding, decrease TREM2-dependent microglia activation and phagocytosis (Schlepckow et al., 2017). It was recently also shown that TREM2 is required for synapse elimination in the developing brain, emphasizing the TREM2-dependent microglia-mediated synaptic refinement (Filipello et al., 2018), which may also play a significant role in the neurodegeneration. Importantly, TREM2 is also a receptor for oligomeric Aβ, and the AD-associated variants in *TREM2* gene (R47H and R62H) compromised the interaction of TREM2 receptor with oligomeric Aβ (Zhao et al., 2018). In the same study, TREM2 was also shown to regulate the catabolism of Aβ in microglia mainly via proteasomal degradation pathways. Apart from this, the role of TREM2 in autophagosomal degradation in microglia was recently highlighted in an AD mouse model (5xFAD), in which TREM2 deficiency impaired mTOR signaling and metabolism in microglia, thus leading to marked induction of autophagy (Ulland et al., 2017). Interestingly, the augmented autophagosomal activity, which is beneficial in terms of reducing inflammation and Aβ load on short term, promoted the formation of dystrophic neurites in *Trem2^-/-^* 5xFAD mice. This suggests that impaired mTOR signaling owing to TREM2 deficiency is harmful and deteriorates the metabolic fitness of microglia in the long term. Consistent with these results, haploinsufficiency of TREM2 in mice impaired the formation of microglial barrier around the Aβ plaques and hence decreased the ability of microglia to envelope Aβ deposits. This reduced compaction of Aβ plaques and increased the total surface area of Aβ fibrils and axonal dystrophy around the plaques (Yuan et al., 2016). Similarly, *Apoe*-deficient mice displayed significantly less compact amyloid plaques together with reduced Aβ plaque-associated microgliosis and the expression of specific DAM genes, as well as increased number of dystrophic neurites around the fibrillar Aβ plaques (Ulrich et al., 2018). Consistent with this, APOE was significantly upregulated in aged mice and mouse models of amyloidosis and tauopathy (Krasemann et al., 2017; Kang et al., 2018). In all of these conditions, a shared set of microglial transcripts were under control of APOE-driven network (Kang et al., 2018). As discussed above, the opposite effects were reported in 5xFAD mice, in which the up-regulation of TREM2 was applied (Figure 2; Lee et al., 2018). However, it should be noted that conflicting data also exist related to the functional outcome measures of TREM2 in mouse models of amyloidosis and tauopathy (Jay et al., 2015; Bemiller et al., 2017; Leyns et al., 2017). Nevertheless, these results provide compelling new evidence that TREM2 acts as a master switch, that regulates the activity and function of microglia in AD and other neurodegenerative diseases.

## The Interplay Between Microglia and Astrocytes Contributes to AD-Associated Synaptotoxicity and Neuroinflammation

In addition to microglia, altered astrocytic activity has been proposed to have negative effects on Aβ clearance and synaptic functions. Among several functions, astrocytes modulate extracellular levels of neurotransmitters by uptake and recycling of glutamate through Na^+^-dependent glutamate transporters (Anderson and Swanson, 2000). Studies utilizing transgenic AD mouse models describe that astrocytes undergo atrophy prior to Aβ plaque accumulation, eventually resulting in deficient glutamatergic transmission, potentially contributing to early stage eNMDAR overstimulation (Olabarria et al., 2010). In addition, astrocytes have been described to reside within the vicinity of Aβ plaques, taking part in the proteolytic clearance of Aβ (Medeiros and LaFerla, 2013). Furthermore, astrocyte activation followed by the release of APOE from astrocytes has been shown to be crucial for the ability of microglia to remove fibrillar Aβ (Terwel et al., 2011). Moreover, exposure to Aβ increases the expression of Aβ-degrading enzymes in astrocytes (Heppner et al., 2015). Irrespective of the described beneficial features of astrocytes, inhibition of astrocyte activation in AD mouse models has been shown to ameliorate AD-like pathology (Furman et al., 2012). This apparent discrepancy may be explained by the fact that activated astrocytes can be subdivided into two categories, A1 and A2 reactive astrocytes (Liddelow et al., 2017; Clarke et al., 2018). More specifically, microglia secreted cytokines (IL-1α, TNF-α, C1q) induce A1 astrocytes, which lack many normal astrocytic functions such as the ability to promote neuronal survival, outgrowth, synaptogenesis and phagocytosis (Figure 1). Moreover, A1 astrocytes induce the death of neurons and oligodendrocytes. Conversely, induction of A2 astrocytes is associated with an increase in neurotrophic factors, suggesting a neuroprotective role. Increased abundance of A1 astrocytes has not only been described in AD, but also in Huntington’s disease, Parkinson’s disease, ALS and MS (Liddelow et al., 2017). Thus, a better understanding of microglial-astrocyte communication and functions for specific astrocyte subtypes is required.

## Novel Genetic Risk Factors Associated With Immune Response and Immune Cells in AD

Research focusing on unraveling the pathogenesis of AD has largely been driven by the amyloid cascade hypothesis, and AD-associated neuroinflammation has been assumed a late-phase response to pathobiological events. Recently, the role of inflammation in AD has been re-evaluated, as GWAS studies have reported several AD-associated genes beyond *TREM2*, including *ABI3*, *CASS4*, *CD33*, *CR1*, *HLA-DRB1/5*, *IL1RAP*, *MEF2C*, *MS4A1*, *MS4A4E*, *PLCγ2*, *SHIP1*, and *PU.1* that are expressed selectively or preferentially in microglia (Heppner et al., 2015; dos Santos et al., 2017; Sims et al., 2017; Bis et al., 2018). Importantly, many of these above-mentioned AD-associated risk genes belong to microglial signaling pathways, which regulate survival, motility, phagocytosis, calcium signaling and gene expression at least partially through TREM2 signaling (Figure 2). Finally, large scale network-based transcriptomic and proteomic studies have identified that microglia as well as immune genes and pathways most strongly associate with the pathophysiology of AD (Zhang et al., 2013; Seyfried et al., 2017). Taken together, these genetic findings suggest a potential role for the immune system in the onset and progression of AD, thus further addressing the involvement of microglia in the early stages of AD pathogenesis.

## Concluding Remarks

The disregard of the potential role of immune system at the early stages of AD has hindered our understanding of AD-associated neuroinflammation. Therefore, recent discoveries related to the underlying molecular mechanisms of neuroinflammation and synaptotoxicity in AD pathogenesis may provide potential novel therapeutic targets and antecedent biomarkers, which are intimately linked to the activity modulation of microglia and astrocyte and their interplay as well as remodeling and engulfment of synapses. The new emerging technologies in single cell analyses, imaging of synaptic and glial functions in humans using novel PET tracers, and novel human-derived cellular models, such as 3D human triculture system to model neurodegeneration and neuroinflammation in AD (Park et al., 2018), will inevitably reinforce the development of new treatment strategies for AD. Furthermore, there are already promising unconventional approaches, such as breaking the immune tolerance by targeting regulatory T-cells to mitigate the AD pathology (Baruch et al., 2015), which will set the basis for other alternative approaches for modulating the onset and progression of AD beyond the conventional Aβ-targeted strategies.

## Author Contributions

MM, MT, AH, and MH designed and outlined the structure and contents of the review. All authors contributed to the literature review, discussion, and writing of the manuscript.

## Conflict of Interest Statement

The authors declare that the research was conducted in the absence of any commercial or financial relationships that could be construed as a potential conflict of interest.

## References

[B1] Abramov E. Dolev I. Fogel H. Ciccotosto G. D. Ruff E. Slutsky I. (2009). Amyloid-beta as a positive endogenous regulator of release probability at hippocampal synapses. *Nat. Neurosci.* 12 1567–1576. 10.1038/nn.2433 19935655

[B2] Anderson C. M. Swanson R. A. (2000). Astrocyte glutamate transport: review of properties, regulation, and physiological functions. *Glia* 32 1–14. 10.1002/1098-1136(200010)32:1<1::AID-GLIA10>3.0.CO;2-W 10975906

[B3] Baruch K. Rosenzweig N. Kertser A. Deczkowska A. Sharif A. M. Spinrad A. (2015). Breaking immune tolerance by targeting Foxp3+ regulatory T cells mitigates Alzheimer’s disease pathology. *Nat. Commun.* 6:7967. 10.1038/ncomms8967 26284939 PMC4557123

[B4] Bemiller S. M. McCray T. J. Allan K. Formica S. V. Xu G. Wilson G. (2017). TREM2 deficiency exacerbates tau pathology through dysregulated kinase signaling in a mouse model of tauopathy. *Mol. Neurodegener.* 12:74. 10.1186/s13024-017-0216-6 29037207 PMC5644120

[B5] Bis J. C. Jian X. Kunkle B. W. Chen Y. Hamilton-Nelson K. L. Bush W. S. (2018). Whole exome sequencing study identifies novel rare and common Alzheimer’s-Associated variants involved in immune response and transcriptional regulation. *Mol. Psychiatry* 10.1038/s41380-018-0112-7 [Epub ahead of print]. 30108311 PMC6375806

[B6] Britschgi M. Takeda-Uchimura Y. Rockenstein E. Johns H. Masliah E. Wyss-Coray T. (2012). Deficiency of terminal complement pathway inhibitor promotes neuronal tau pathology and degeneration in mice. *J. Neuroinflamm.* 9:722. 10.1186/1742-2094-9-220 22989354 PMC3511294

[B7] Cirrito J. R. Kang J.-E. Lee J. Stewart F. R. Verges D. K. Silverio L. M. (2008). Endocytosis is required for synaptic activity-dependent release of amyloid-beta in vivo. *Neuron* 58 42–51. 10.1016/j.neuron.2008.02.003 18400162 PMC2390913

[B8] Clarke L. E. Liddelow S. A. Chakraborty C. Münch A. E. Heiman M. Barres B. A. (2018). Normal aging induces A1-like astrocyte reactivity. *Proc. Natl. Acad. Sci.* 115 E1896–E1905. 10.1073/pnas.1800165115 29437957 PMC5828643

[B9] Coleman P. D. Yao P. J. (2003). Synaptic slaughter in Alzheimer’s disease. *Neurobiol. Aging* 24 1023–1027. 10.1016/j.neurobiolaging.2003.09.001 14643374

[B10] Danysz W. Parsons C. G. Mobius H. J. Stoffler A. Quack G. (2000). Neuroprotective and symptomatological action of memantine relevant for Alzheimer’s disease–a unified glutamatergic hypothesis on the mechanism of action. *Neurotox. Res.* 2 85–97. 10.1007/BF03033787 16787834

[B11] De Strooper B. Karran E. (2016). The cellular phase of alzheimer’s disease. *Cell* 164 603–615. 10.1016/j.cell.2015.12.056 26871627

[B12] Decker H. Lo K. Y. Unger S. M. Ferreira S. T. Silverman M. A. (2010). Amyloid- peptide oligomers disrupt axonal transport through an NMDA receptor-dependent mechanism that is mediated by glycogen synthase kinase 3 in primary cultured hippocampal neurons. *J. Neurosci.* 30 9166–9171. 10.1523/JNEUROSCI.1074-10.2010 20610750 PMC6632489

[B13] Deczkowska A. Amit I. Schwartz M. (2018). Microglial immune checkpoint mechanisms. *Nat. Neurosci.* 21 779–786. 10.1038/s41593-018-0145-x 29735982

[B14] dos Santos L. R. Pimassoni L. H. S. Sena G. G. S. Camporez D. Belcavello L. Trancozo M. (2017). Validating GWAS variants from microglial genes implicated in alzheimer’s disease. *J. Mol. Neurosci.* 62 215–221. 10.1007/s12031-017-0928-7 28477215

[B15] El Khoury J. B. Moore K. J. Means T. K. Leung J. Terada K. Toft M. (2003). CD36 mediates the innate host response to β-amyloid. *J. Exp. Med.* 197 1657–1666. 10.1084/jem.20021546 12796468 PMC2193948

[B16] Falcon S. Gentleman R. (2007). Using GOstats to test gene lists for GO term association. *Bioinformatics* 23 257–258. 10.1093/bioinformatics/btl567 17098774

[B17] Ferreira I. L. Bajouco L. M. Mota S. I. Auberson Y. P. Oliveira C. R. Rego A. C. (2012). Amyloid beta peptide 1–42 disturbs intracellular calcium homeostasis through activation of GluN2B-containing N-methyl-d-aspartate receptors in cortical cultures. *Cell Calcium* 51 95–106. 10.1016/j.ceca.2011.11.008 22177709

[B18] Filipello F. Morini R. Corradini I. Zerbi V. Canzi A. Michalski B. (2018). The microglial innate immune receptor TREM2 is required for synapse elimination and normal brain connectivity. *Immunity* 48 979.e8–991.e8. 10.1016/j.immuni.2018.04.016 29752066

[B19] Frandemiche M. L. De Seranno S. Rush T. Borel E. Elie A. Arnal I. (2014). Activity-dependent tau protein translocation to excitatory synapse is disrupted by exposure to amyloid-beta oligomers. *J. Neurosci.* 34 6084–6097. 10.1523/JNEUROSCI.4261-13.2014 24760868 PMC6608293

[B20] Furman J. L. Sama D. M. Gant J. C. Beckett T. L. Murphy M. P. Bachstetter A. D. (2012). Targeting astrocytes ameliorates neurologic changes in a mouse model of alzheimer’s disease. *J. Neurosci.* 32 16129–16140. 10.1523/JNEUROSCI.2323-12.2012 23152597 PMC3506017

[B21] Guntupalli S. Widagdo J. Anggono V. (2016). Amyloid-β-induced dysregulation of AMPA receptor trafficking. *Neural Plast.* 2016:3204519. 10.1155/2016/3204519 27073700 PMC4814684

[B22] Hardingham G. E. Bading H. (2010). Synaptic versus extrasynaptic NMDA receptor signalling: implications for neurodegenerative disorders. *Nat. Rev. Neurosci.* 11 682–696. 10.1038/nrn2911 20842175 PMC2948541

[B23] Heppner F. L. Ransohoff R. M. Becher B. (2015). Immune attack: the role of inflammation in Alzheimer disease. *Nat. Rev. Neurosci.* 16 358–372. 10.1038/nrn3880 25991443

[B24] Hong S. Beja-Glasser V. F. Nfonoyim B. M. Frouin A. Li S. Ramakrishnan S. (2016). Complement and microglia mediate early synapse loss in Alzheimer mouse models. *Science* 352 712–716. 10.1126/science.aad8373 27033548 PMC5094372

[B25] Ittner A. Chua S. W. Bertz J. Volkerling A. van der Hoven J. Gladbach A. (2016). Site-specific phosphorylation of tau inhibits amyloid-β toxicity in Alzheimer’s mice. *Science* 354 904–908. 10.1126/science.aah6205 27856911

[B26] Ittner L. M. Ke Y. D. Delerue F. Bi M. Gladbach A. van Eersel J. (2010). Dendritic function of tau mediates amyloid-β toxicity in alzheimer’s disease mouse models. *Cell* 142 387–397. 10.1016/J.CELL.2010.06.036 20655099

[B27] Jay T. R. Miller C. M. Cheng P. J. Graham L. C. Bemiller S. Broihier M. L. (2015). TREM2 deficiency eliminates TREM2 + inflammatory macrophages and ameliorates pathology in Alzheimer’s disease mouse models. *J. Exp. Med.* 212 287–295. 10.1084/jem.20142322 25732305 PMC4354365

[B28] Jonsson T. Atwal J. K. Steinberg S. Snaedal J. Jonsson P. V. Bjornsson S. (2012). A mutation in APP protects against Alzheimer‘s disease and age-related cognitive decline. *Nature* 488:96. 10.1038/nature11283 22801501

[B29] Kang S. S. Ebbert M. T. W. Baker K. E. Cook C. Wang X. Sens J. P. (2018). Microglial translational profiling reveals a convergent APOE pathway from aging, amyloid, and tau. *J. Exp. Med.* 215 2235–2245. 10.1084/jem.20180653 30082275 PMC6122978

[B30] Keren-shaul H. Spinrad A. Weiner A. Colonna M. Schwartz M. Amit I. (2017). A unique microglia type associated with restricting development of alzheimer ’ s disease article a unique microglia type associated with restricting development of alzheimer’s disease. *Cell* 169 1–15. 10.1016/j.cell.2017.05.018 28602351

[B31] Kolev M. V. Ruseva M. M. Harris C. L. Morgan B. P. Donev R. M. (2009). Implication of complement system and its regulators in Alzheimer’s disease. *Curr. Neuropharmacol.* 7 1–8. 10.2174/157015909787602805 19721814 PMC2724661

[B32] Krasemann S. Madore C. Cialic R. Baufeld C. Calcagno N. El Fatimy R. (2017). The TREM2-APOE pathway drives the transcriptional phenotype of dysfunctional microglia in neurodegenerative diseases. *Immunity* 47 566.e9–581.e9. 10.1016/j.immuni.2017.08.008 28930663 PMC5719893

[B33] Lazarevic V. Fieǹko S. Andres-Alonso M. Anni D. Ivanova D. Montenegro-Venegas C. (2017). Physiological concentrations of amyloid beta regulate recycling of synaptic vesicles via alpha7 acetylcholine receptor and CDK5/calcineurin signaling. *Front. Mol. Neurosci.* 10:221. 10.3389/fnmol.2017.00221 28785201 PMC5520466

[B34] Lee C. Y. D. Daggett A. Gu X. Jiang L.-L. Langfelder P. Li X. (2018). Elevated TREM2 gene dosage reprograms microglia responsivity and ameliorates pathological phenotypes in alzheimer’s disease models. *Neuron* 97 1032.e5–1048.e5. 10.1016/j.neuron.2018.02.002 29518357 PMC5927822

[B35] Leyns C. E. G. Ulrich J. D. Finn M. B. Stewart F. R. Koscal L. J. Remolina Serrano J. (2017). TREM2 deficiency attenuates neuroinflammation and protects against neurodegeneration in a mouse model of tauopathy. *Proc. Natl. Acad. Sci.* 114 11524–11529. 10.1073/pnas.1710311114 29073081 PMC5663386

[B36] Li S. Jin M. Koeglsperger T. Shepardson N. E. Shankar G. M. Selkoe D. J. (2011). Soluble a oligomers inhibit long-term potentiation through a mechanism involving excessive activation of extrasynaptic NR2B-containing NMDA receptors. *J. Neurosci.* 31 6627–6638. 10.1523/JNEUROSCI.0203-11.2011 21543591 PMC3100898

[B37] Liddelow S. A. Guttenplan K. A. Clarke L. E. Bennett F. C. Bohlen C. J. Schirmer L. (2017). Neurotoxic reactive astrocytes are induced by activated microglia. *Nature* 541 481–487. 10.1038/nature21029 28099414 PMC5404890

[B38] Lüscher C. Malenka R. C. (2012). NMDA receptor-dependent long-term potentiation and long-term depression (LTP/LTD). *Cold Spring Harb. Perspect. Biol.* 4:a005710. 10.1101/cshperspect.a005710 22510460 PMC3367554

[B39] Martiskainen H. Herukka S. K. Stančáková A. Paananen J. Soininen H. Kuusisto J. (2017). Decreased plasma β-amyloid in the Alzheimer’s disease APP A673T variant carriers. *Ann. Neurol.* 82 128–132. 10.1002/ana.24969 28556232

[B40] Matos M. Augusto E. Oliveira C. R. Agostinho P. (2008). Amyloid-beta peptide decreases glutamate uptake in cultured astrocytes: involvement of oxidative stress and mitogen-activated protein kinase cascades. *Neuroscience* 156 898–910. 10.1016/j.neuroscience.2008.08.022 18790019

[B41] Medeiros R. LaFerla F. M. (2013). Astrocytes: conductors of the alzheimer disease neuroinflammatory symphony. *Exp. Neurol.* 239 133–138. 10.1016/j.expneurol.2012.10.007 23063604

[B42] Miller K. R. Streit W. J. (2007). The effects of aging, injury and disease on microglial function: a case for cellular senescence. *Neuron Glia Biol.* 3 245–253. 10.1017/S1740925X08000136 18634615

[B43] Olabarria M. Noristani H. N. Verkhratsky A. Rodríguez J. J. (2010). Concomitant astroglial atrophy and astrogliosis in a triple transgenic animal model of Alzheimer’s disease. *Glia* 58 831–838. 10.1002/glia.20967 20140958

[B44] Paolicelli R. C. Bolasco G. Pagani F. Maggi L. Scianni M. Panzanelli P. (2011). Synaptic pruning by microglia is necessary for normal brain development. *Science* 333 1456–1458. 10.1126/science.1202529 21778362

[B45] Parihar M. S. Brewer G. J. (2010). Amyloid-β as a modulator of synaptic plasticity. *J. Alzheimers Dis.* 22 741–763. 10.3233/JAD-2010-101020 20847424 PMC3079354

[B46] Park J. Wetzel I. Marriott I. Dréau D. D’Avanzo C. Kim D. Y. (2018). A 3D human triculture system modeling neurodegeneration and neuroinflammation in Alzheimer’s disease. *Nat. Neurosci.* 21 941–951. 10.1038/s41593-018-0175-4 29950669 PMC6800152

[B47] Prince M. Comas-Herrera M. A. Knapp M. Guerchet M. Karagiannidou M. M. (2016). *World Alzheimer Report 2016 Improving Healthcare for People Living with Dementia Coverage, QualIty and Costs Now and In the Future.* London: Alzheimer’s Disease International.

[B48] Rönicke R. Mikhaylova M. Rönicke S. Meinhardt J. Schröder U. H. Fändrich M. (2011). Early neuronal dysfunction by amyloid β oligomers depends on activation of NR2B-containing NMDA receptors. *Neurobiol. Aging* 32 2219–2228. 10.1016/J.NEUROBIOLAGING.2010.01.011 20133015

[B49] Scheltens P. Blennow K. Breteler M. M. B. De Strooper B. Frisoni G. B. Salloway S. (2016). *Alzheimer’s Disease.* Available at: www.thelancet.com. 10.1016/S0140-6736(15)01124-1 26921134

[B50] Schlepckow K. Kleinberger G. Fukumori A. Feederle R. Lichtenthaler S. F. Steiner H. (2017). An Alzheimer-associated TREM2 variant occurs at the ADAM cleavage site and affects shedding and phagocytic function. *EMBO Mol. Med.* 9 1356–1365. 10.15252/emmm.201707672 28855300 PMC5623859

[B51] Seyfried N. T. Dammer E. B. Swarup V. Nandakumar D. Duong D. M. Yin L. (2017). A multi-network approach identifies protein-specific co-expression in asymptomatic and symptomatic alzheimer’s disease. *Cell Syst.* 4 60.e4–72.e4. 10.1016/j.cels.2016.11.006 27989508 PMC5269514

[B52] Shankar G. M. Bloodgood B. L. Townsend M. Walsh D. M. Selkoe D. J. Sabatini B. L. (2007). Natural oligomers of the alzheimer amyloid- protein induce reversible synapse loss by modulating an NMDA-type glutamate receptor-dependent signaling pathway. *J. Neurosci.* 27 2866–2875. 10.1523/JNEUROSCI.4970-06.2007 17360908 PMC6672572

[B53] Shi Q. Chowdhury S. Ma R. Le K. X. Hong S. Caldarone B. J. (2017). Complement C3 deficiency protects against neurodegeneration in aged plaque-rich APP/PS1 mice. *Sci. Transl. Med.* 9:eaaf6295. 10.1126/scitranslmed.aaf6295 28566429 PMC6936623

[B54] Sims R. van der Lee S. J. Naj A. C. Bellenguez C. Badarinarayan N. Jakobsdottir J. (2017). Rare coding variants in PLCG2, ABI3, and TREM2 implicate microglial-mediated innate immunity in Alzheimer’s disease. *Nat. Genet.* 49 1373–1384. 10.1038/ng.3916 28714976 PMC5669039

[B55] Snyder E. M. Nong Y. Almeida C. G. Paul S. Moran T. Choi E. Y. (2005). Regulation of NMDA receptor trafficking by amyloid-β. *Nat. Neurosci.* 8 1051–1058. 10.1038/nn1503 16025111

[B56] Stephan A. H. Barres B. A. Stevens B. (2012). The complement system: an unexpected role in synaptic pruning during development and disease. *Annu. Rev. Neurosci.* 35 369–389. 10.1146/annurev-neuro-061010-113810 22715882

[B57] Stewart C. R. Stuart L. M. Wilkinson K. Van Gils J. M. Deng J. Halle A. (2010). CD36 ligands promote sterile inflammation through assembly of a Toll-like receptor 4 and 6 heterodimer. *Nat. Immunol.* 11 155–161. 10.1038/ni.1836 20037584 PMC2809046

[B58] Suarez-Calvet M. Araque Caballero M. A. Kleinberger G. Bateman R. J. Fagan A. M. Morris J. C. (2016). Early changes in CSF sTREM2 in dominantly inherited Alzheimers disease occur after amyloid deposition and neuronal injury. *Sci. Transl. Med.* 8:369ra178. 10.1126/scitranslmed.aag1767 27974666 PMC5385711

[B59] Tackenberg C. Grinschgl S. Trutzel A. Santuccione A. C. Frey M. C. Konietzko U. (2013). NMDA receptor subunit composition determines beta-amyloid-induced neurodegeneration and synaptic loss. *Cell Death Dis.* 4:e608. 10.1038/cddis.2013.129 23618906 PMC3641351

[B60] Terwel D. Steffensen K. R. Verghese P. B. Kummer M. P. Gustafsson J.-A. Holtzman D. M. (2011). Critical role of astroglial apolipoprotein E and liver X receptor- expression for microglial A phagocytosis. *J. Neurosci.* 31 7049–7059. 10.1523/JNEUROSCI.6546-10.2011 21562267 PMC6703224

[B61] Tu S. Okamoto S. Lipton S. A. Xu H. (2014). Oligomeric Aβ-induced synaptic dysfunction in Alzheimer’s disease. *Mol. Neurodegener.* 9:48. 10.1186/1750-1326-9-48 25394486 PMC4237769

[B62] Ulland T. K. Song W. M. Huang S. C. C. Ulrich J. D. Sergushichev A. Beatty W. L. (2017). TREM2 maintains microglial metabolic fitness in alzheimer’s disease. *Cell* 170 649.e13–663.e13. 10.1016/j.cell.2017.07.023 28802038 PMC5573224

[B63] Ulrich J. D. Ulland T. K. Mahan T. E. Nyström S. Nilsson K. P. Song W. M. (2018). ApoE facilitates the microglial response to amyloid plaque pathology. *J. Exp. Med.* 215 1047–1058. 10.1084/jem.20171265 29483128 PMC5881464

[B64] Van Der Kant R. Goldstein L. S. B. (2015). Cellular functions of the amyloid precursor protein from development to dementia. *Dev. Cell* 32 502–515. 10.1016/j.devcel.2015.01.022 25710536

[B65] Venegas C. Kumar S. Franklin B. S. Dierkes T. Brinkschulte R. Tejera D. (2017). Microglia-derived ASC specks cross-seed amyloid-β in Alzheimer’s disease. *Nature* 552 355–361. 10.1038/nature25158 29293211

[B66] Yeh F. L. Wang Y. Tom I. Gonzalez L. C. Sheng M. (2016). TREM2 binds to apolipoproteins, including APOE and CLU/APOJ, and thereby facilitates uptake of amyloid-beta by microglia. *Neuron* 91 328–340. 10.1016/j.neuron.2016.06.015 27477018

[B67] Yuan P. Condello C. Keene C. D. Wang Y. Bird T. D. Paul S. M. (2016). TREM2 haplodeficiency in mice and humans impairs the microglia barrier function leading to decreased amyloid compaction and severe axonal dystrophy. *Neuron* 92 252–264. 10.1016/j.neuron.2016.09.016 27710785

[B68] Zhai Q. Li F. Chen X. Jia J. Sun S. Zhou D. (2017). Triggering receptor expressed on myeloid cells 2, a novel regulator of immunocyte phenotypes, confers neuroprotection by relieving neuroinflammation. *Anesthesiology* 127 98–110. 10.1097/ALN.0000000000001628 28398927

[B69] Zhang B. Gaiteri C. Bodea L. G. Wang Z. McElwee J. Podtelezhnikov A. A. (2013). Integrated systems approach identifies genetic nodes and networks in late-onset Alzheimer’s disease. *Cell* 153 707–720. 10.1016/j.cell.2013.03.030 23622250 PMC3677161

[B70] Zhang Y. Thompson R. Zhang H. Xu H. (2011). APP processing in Alzheimer’s disease. *Mol. Brain* 4:3. 10.1186/1756-6606-4-3 21214928 PMC3022812

[B71] Zhao Y. Wu X. Li X. Jiang L. L. Gui X. Liu Y. (2018). TREM2 is a receptor for -amyloid that mediates microglial function. *Neuron* 97 1023.e7–1031.e7. 10.1016/j.neuron.2018.01.031 29518356 PMC5889092

